# Synergy between intention recognition and commitments in cooperation dilemmas

**DOI:** 10.1038/srep09312

**Published:** 2015-03-20

**Authors:** The Anh Han, Francisco C. Santos, Tom Lenaerts, Luís Moniz Pereira

**Affiliations:** 1 School of Computing, Teesside University, Borough Road, Middlesbrough, TS1 3BA, UK; 2 INESC-ID and Instituto Superior Técnico, Universidade de Lisboa, IST-Taguspark, 2744-016 Porto Salvo, Portugal; 3 ATP-group, Instituto para a Investigação Interdisciplinar, P-1649-003 Lisboa Codex, Portugal; 4 AI lab, Computer Science Department, Vrije Universiteit Brussel, Pleinlaan 2, 1050 Brussels, Belgium; 5 MLG, Département d'Informatique, Université Libre de Bruxelles, Boulevard du Triomphe CP212, 1050 Brussels, Belgium; 6 NOVA Laboratory for Computer Science and Informatics (NOVA LINCS), Departamento de Informática, Faculdade de Ciências e Tecnologia, Universidade Nova de Lisboa, 2829-516 Caparica, Portugal

## Abstract

Commitments have been shown to promote cooperation if, on the one hand, they can be sufficiently enforced, and on the other hand, the cost of arranging them is justified with respect to the benefits of cooperation. When either of these constraints is not met it leads to the prevalence of commitment free-riders, such as those who commit only when someone else pays to arrange the commitments. Here, we show how intention recognition may circumvent such weakness of costly commitments. We describe an evolutionary model, in the context of the one-shot Prisoner's Dilemma, showing that if players first predict the intentions of their co-player and propose a commitment only when they are not confident enough about their prediction, the chances of reaching mutual cooperation are largely enhanced. We find that an advantageous synergy between intention recognition and costly commitments depends strongly on the confidence and accuracy of intention recognition. In general, we observe an intermediate level of confidence threshold leading to the highest evolutionary advantage, showing that neither unconditional use of commitment nor intention recognition can perform optimally. Rather, our results show that arranging commitments is not always desirable, but that they may be also unavoidable depending on the strength of the dilemma.

Since Darwin, the problem of explaining the evolution of cooperative behavior has been actively investigated in many fields, from Evolutionary Biology, Ecology, to Economics and Social Science. Several mechanisms responsible for the evolution of cooperation have been proposed, from kin and group selection to direct and indirect reciprocity, to structured population, and to punishment^1^,^2^,^3^,^4^,^5^. Recently, a large body of economic experiments and theoretical studies have shown that high levels of cooperation can be achieved if reliable agreements can be arranged^6^,^7^,^8^,^9^,^10^,^11^,^12^,^13^,^14^. Arranging prior commitments, such as through enforceable contracts or pledges^8^, deposit-refund scheme^11^,^12^ or even emotional or reputation-based commitment devices^7^,^9^, provides incentives for others to cooperate, clarifying the preferences or intentions of others^8^,^15^,^16^. However, in human societies, not all cooperative ventures require explicit prior commitments to be made. On the one hand, arranging reliable commitments may be very costly (and take time)^15^, which can lead to the prevalence of commitment free-riders, and, on the other hand, others' intentions might be clarified without using a commitment device. Contracts are a popular kind of commitment, which play a key role in enforcing cooperation in modern societies. But even then people occasionally prefer not to rely on using a contract, as are the cases for interactions between relatives or close friends, or between (or with) trustworthy brands. In such cases, partners' cooperative behavior can be envisaged with high confidence. People also do not ask for promise or making threats when partners' motivations can be predicted with high confidence, as doing so may lead to negative reactions or an implication of distrust from them^13^,^17^.

Additionally, human beings are experts in mind reading, particularly at discerning what others are perceiving and intending^18^. An ability to assess intention in others, which is clearly possessed by humans^19^,^20^, has been demonstrated to play a promoting role for the emergence of cooperation. It enables individuals to assess cooperative intention in others in noisy and uncertain environments, and to identify those with an exploitative intent^8^,^16^,^21^,^22^,^23^. In addition, behavioral experiments show that people do care about and distinguish between real intentions and outcomes, and that difference plays a crucial role in their decision, for instance, whether to cooperate or to defect, and to reward or to punish^21^,^24^,^25^,^26^. Although recognizing an intention cannot always be done with high enough confidence to make any decision based on it, an ability to assess intention in others, based on previous experience and available observations at hand, allows choosing cooperative partners even without resorting to commitment devices.

Thus motivated, here we investigate whether a conditional use of commitment through intention recognition can promote the emergence of cooperation in the one-shot Prisoner's Dilemma. In its simple form, a cooperative act (C) is to pay a cost (*c*) for its co-player to receive a benefit (*b* > *c*), while a defective act (D) is to spend nothing and thus provides its co-player with no benefit. In a one-shot pairwise interaction, for each player it is better to play D, leading to a zero payoff for both, while both can obtain a higher payoff (*b*–*c*) if they simultaneously choose C. Here, we consider a strategy, which, at each interaction, attempts first to assess the co-player's intention (whether to cooperate or to defect). Only when it is not confident about what the co-player intends to do in the current interaction, does it propose to the co-player a commitment deal. A commitment proposer pays a cost of arrangement (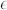) to make the commitment credible, but those who commit but then default have to provide the co-player with a compensation (*δ*)^27^. It has been shown^11^,^12^,^14^,^27^, that substantial levels of cooperation are achieved if both the cost of arranging commitment is small enough compared to the cost of cooperation, and a sufficiently high compensation can be enforced. However, if either of these two conditions is not satisfied, commitment free-riders can take over and become dominant^27^. On the one hand, if the cost of arranging commitment is too large, those who commit and cooperate only if someone else pays to arrange the commitment for them are dominant. On the other hand, when the cost of compensation is too low, for instance due to the difficulty of enforcing the deal afterwards, those who agree on the commitment but then default on it dominate the commitment proposers.

We show that a conditional use of commitments, by means of first assessing intentions of the co-player, can facilitate the commitment free-riding issue, ameliorating the performance of commitment and leading to improved cooperation. The key parameter in our model is a *confidence threshold (θ)*, which is utilized to decide when intention recognition can be relied on (to choose a move), or a commitment deal needs to be arranged to clarify the co-player's intention. The questions we would like to ask here are whether such a conditional use of commitment can resolve the commitment free-riding issues, particularly when a strong commitment cannot be arranged. Furthermore, what is the appropriate confidence threshold, inasmuch the benefit and the cost of commitments and the accuracy of the intention recognition vary?

## Results

We consider here, next to the traditional pure cooperator (C) and defector (D) strategies, a new strategy which combines intention recognition and commitment arrangement, denoted by IRCOM. In an interaction, IRCOM recognizes the intention (to cooperate or to defect) of its co-player. A confidence level, *x* ∈ [0, 1], is assigned to the recognition result. It defines the degree of confidence, in terms of a probability, that IRCOM predicts the co-player's intention correctly. Then, if it is confident enough about the prediction, that is if *x* is greater than a given, so-called, *confidence threshold*, *θ* ∈ [0, 1], then in the current interaction it cooperates if the recognized intention of the co-player is to cooperate, and defects otherwise.

When IRCOM is not sufficiently confident about its co-player's intention, i.e. *x* < *θ*, it proposes a commitment to others and subsequently cooperates if the opponent accepts the deal. If the deal is not accepted, then this IRCOM refuses to play the game. We consider two additional commitment free-riding strategies^14^,^27^: (i) The fake committers (FAKE), who accept a commitment proposal yet defect when playing the game, presuming that they can exploit the commitment proposers without suffering a severe consequence; and, (ii) the commitment free-riders (FREE), who defect unless being proposed a commitment, which they then accept and next cooperate in the PD game. In other words, these players are willing to cooperate when a commitment is arranged but are not prepared to pay the cost of setting it up.

However, the prediction being made can be wrong. We assume that prediction accuracy and confidence are positively correlated^28^,^29^,^30^. Namely, the probability of a correct prediction is, *y* = *r* × *x*, where *r* > 0 is dubbed the *accuracy-to-confidence* ratio. Assuming that the confidence, *x*, is uniformly distributed in [0, 1], the payoff matrix for IRCOM reads
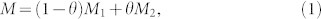
where *M*_1_ and *M*_2_ are the payoff matrices when IRCOM plays without proposing a commitment (i.e. when *x* > *θ*) and when it does so (i.e. when *x* ≤ *θ*), respectively. For details of the computation of the two matrices see Methods and Supporting Information (SI). Table 1 summarizes the parameters and variables in our model.

Note that if *x* ≤ *θ*, i.e. IRCOM is not confident enough about its intention prediction, it behaves the same as a pure commitment proposer (COMP)^27^ when interacting with the non-proposing commitment strategies (i.e. C, D, FAKE and FREE). The greater *θ* is, the more cautious IRCOM is about its intention recognition result, thereby tending to use commitments more frequently. In an interaction between IRCOM and COMP, we consider that COMP always proposes first and pays the arrangement cost 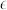 due to the time delay and effort IRCOM spends on intention recognition deliberation.

### Emergence of conditional commitment and cooperation

**Figure 1:**
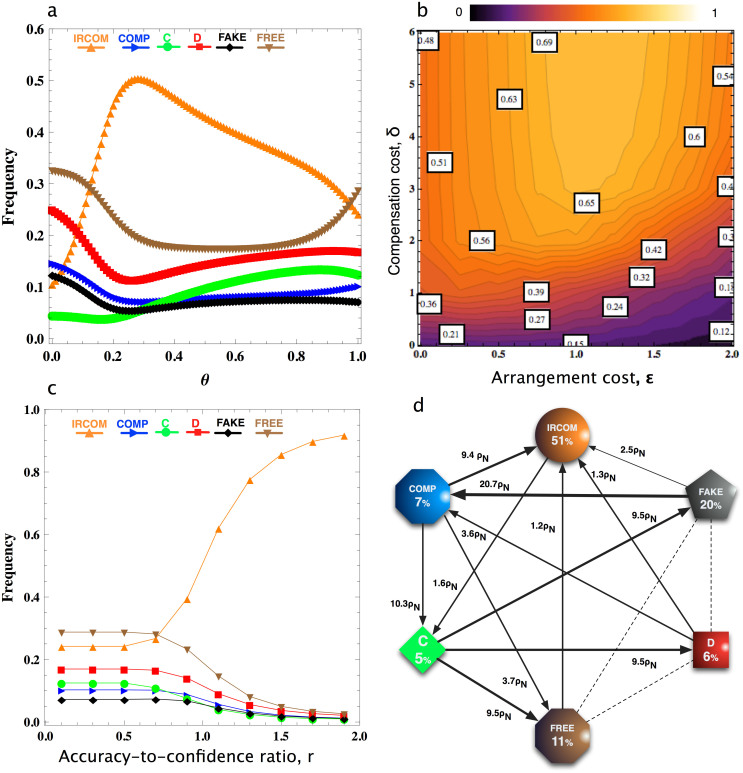
(a) Frequency of each strategy as a function of confidence threshold *θ*. In a population of IRCOM, COMP, C, D, FAKE and FREE individuals, for a sufficiently large *θ*, IRCOM is most frequent in the population. The performance of IRCOM decreases when *θ* is too high. It implies that IRCOM should not be too cautious about its intention recognition capacity, i.e. not be too careful to always propose commitment instead of believing in its prediction accuracy; (b) Frequency of IRCOM at the optimal confidence threshold, as a function of the cost of arranging commitment 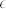 and the compensation cost *δ*. Interestingly, in contrast to COMP, it is not always the case that the frequency of IRCOM is smaller for larger 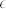. IRCOM is actually more frequent when 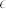 is sufficiently large. (c) Frequency of each strategy as a function of accuracy to confidence ratio, *r*, at the optimal confidence threshold. When intention recognition accuracy is sufficiently high, IRCOM is prevalent, but when it is small, FREE is most abundant. (d) Transitions probabilities and stationary distributions (*θ* = 0.28). Note the transitions from COMP to FREE to IRCOM. For clarity, only the transitions that are larger than neutral are shown (*ρ_N_* = 1/*N* denotes the neutral transition probability). Parameters: In panels (a), (c) and (d): *δ* = 4; 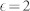; In panels (a), (b) and (d): *r* = 1; In all cases, *b* = 4; *c* = 1; *N* = 100; *β* = 0.1.

We first study the stationary distribution in a population of the six above described strategies, namely IRCOM, COMP, C, D, FAKE and FREE (see Methods). The results show that, for a large range of the confidence threshold *θ*, IRCOM is dominant, whereas the population spends most of the time in the homogenous state of IRCOM, regardless of the initial composition of the population (Figure 1a). However, when *θ* is low, free-riding strategies become dominant. That is, when IRCOM does not have sufficient confidence about whether its co-player intends to cooperate or to defect in the current interaction, it would be better off counting on arranging a (costly) commitment deal.

Figure 1b shows that the prevalence of IRCOM endures for a wide range of 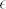 and *δ*, as long as an appropriate *θ* is adopted. Interestingly, in contrast to COMP^27^, it is not always the case that the frequency of IRCOM is demolished when 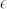 increases (see also Figure S2 in SI). IRCOM actually becomes more frequent when 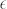 is sufficiently high, but not too high. This is mainly because IRCOM suppresses the commitment free-riders for a wider range of 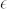, as can be seen from Figure 1d where we show the transition probabilities and the transition directions amongst the six strategies. Namely, for a sufficiently high 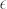 (namely, 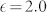), COMP is taken over by the FREE players, against which IRCOM still is a viable strategy. However, when 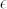 is too large, IRCOM is again taken over by FREE players (see Figure S4 in the SI for a larger 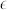). The viability of IRCOM in dealing with commitment free-riders is robust for varying the accuracy-to-confidence ratio, *r*, as shown in Figure 1c. Namely, we observe that IRCOM is the dominant strategy whenever this ratio is sufficiently high, although the commitment free-riding strategy FREE takes over when *r* is too small. That is, whenever intention recognition can be performed with a sufficiently high accuracy, as are the case for instance in repeated games^16^,^23^ or when the intention recognition process is facilitated^21^,^26^, IRCOM is amply sufficient at dealing with commitment free-riders.

**Figure 2:**
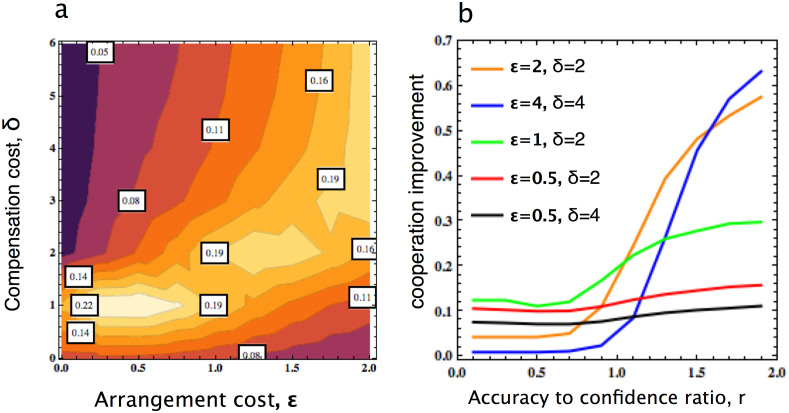
(a) Improvement in cooperation level obtained from IRCOM and COMP compared to the case where there is no IRCOM, as a function of the cost of arranging commitment 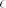 and the compensation cost *δ*. Improvement is achieved for a wide range of 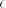 and *δ*. It is most significant when 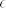 is rather high and *δ* is not too large, i.e. the commitment deal is weak (see Figure S1 in SI for the improvement obtained in percentage, and also for other parameter values). (b) Such improvement as a function of the accuracy-to-confidence ration, *r*, and for different commitment deals. In general, the larger *r*, the more significant improvement is obtained. Furthermore, when *r* is sufficiently high, larger improvement is obtained when it is costly to arrange commitments and/or a high compensation is difficult to enforced. Parameters: *b* = 4, *c* = 1, *N* = 100, and *β* = 0.1. In panel (a), r = 1.

We now analyze whether and when the conditional use of commitment can actually facilitate the evolution of cooperation. To that end, we make a direct comparison in terms of the level of cooperation obtained through commitment strategies in our model, i.e. from IRCOM and COMP, and such a level in the unconditional commitment model where IRCOM is not included, see Figure 2. The results show that certain improvement is possible for a wide range of commitment deals, i.e. for varying 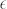 and *δ*, see Figure 2a. Interestingly, the improvement is most significant when the commitment deal is weak, that is, when it is rather costly to arrange (high 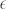) and/or no sufficiently high compensation can be enforced (low *δ*). It is exactly when COMP does not perform well, as it is dominated by the commitment free-riders FREE and FAKE in either condition (i.e. high 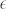 or low *δ*), respectively^27^. This notable observation is robust for varying *r*, as can be seen in Figure 2b: the improvement in terms of cooperation is positive in general, and increases with *r*. Furthermore, the improvement is substantial for large 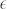 (see for instance cases with 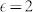 and 4). In SI, we show that the improvement is also more significant when the benefit-to-cost ratio is larger (see Figure S1).

**Figure 3:**
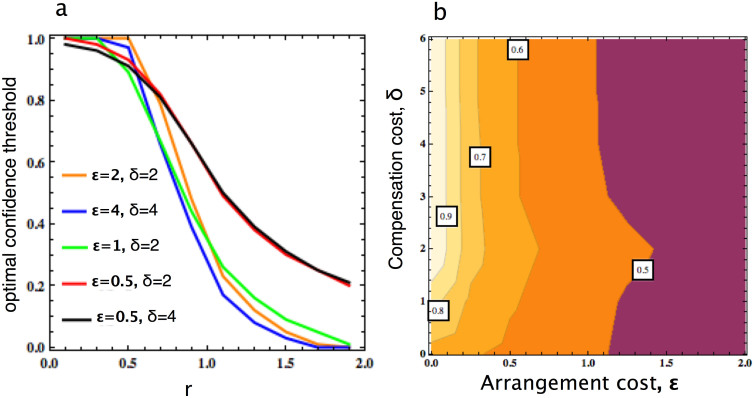
Optimal confidence threshold, (a) as a function of *r*, for different commitment deals, and (b) as a function of 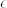 and *δ*. In general, the higher *r* and the larger 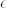, the lower confidence level needs to be attained to rely on intention recognition predictions (i.e. taking higher risk). This confidence level does not significantly depend on *δ*. We adopt, in both cases, *b* = 4, *c* = 1, *N* = 100, and *β* = 0.1. In panel (b), *r* = 1.

We now ask, when should one take more risk, avoiding to arrange costly commitment? In Figure 3 we address the effect of varying 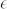 and *δ*, as well as varying the accuracy over confidence ratio *r*. In general, the higher 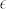 and the higher *r*, the lower confidence level needs to be attained to rely on intention recognition predictions. That is, as the PD becomes more beneficial and the intention recognition prediction can be carried out more accurately, a smaller confidence is exacted to rely on intention recognition, thereby avoiding the cost of arranging commitment. We also observe that this confidence level does not significantly depend on *δ*, see Figure 3b.

## Discussion

We have shown, within the context of the one-shot Prisoner's Dilemma (PD), that a conditional use of commitment based on a subjective confidence in assessing a co-player's intention can lead to improved levels of commitment and cooperation. In general, by avoiding the payment of the cost of arranging commitments whenever gaining a sufficient confidence about the co-player's intention, an evolutionary advantage can be achieved. Waiting for a too large confidence may lead to unnecessarily paying the cost, though it can be avoided. However, doing so when confidence is low allows defectors and commitment free-riders to exploit, leading to the destruction of cooperation. Our results show that the gained improvement via the intention recognition capability is more significant when the PD is less harsh, and as more accurate predictions can be achieved. Interestingly, such an improvement is most significant when the cost of arranging commitments is high, thereby overcoming the weaker cases of using the pure commitment strategy^27^. Moreover, our analysis suggests that, as the PD becomes more beneficial and the prediction is more accurate, a smaller confidence is required to enable one to take the risk involved in avoiding to arrange costly commitments. These results suggest that, although many societies may have evolved mechanisms to facilitate the making and the enforcement of prior commitments (e.g. legal contracts)^9^,^15^, the cost-efficiency problem faced when implementing such mechanisms (e.g. law systems) may be coped with by using more complex cognitive skills such as of intention recognition (which has been demonstrated to be prevalent in humans and primates^18^,^19^,^20^), in order to facilitate further the sustainability of the commitment mechanisms, hence cooperation.

Our results are in line with the work in Ref. 31, where a resource claiming model is described. In that model, players can choose whether to engage in a fight for a resource based on their estimation of the opponents' capability and the players' confidence about their own capacity. It has been shown that overconfidence (which is equivalent to the avoidance of arranging costly commitment at a low confidence threshold in our model) can become evolutionarily stable when the resource is sufficiently large compared to the cost of fighting, as the players might lose their chance of winning the resource if not being confident enough even when they have a stronger capacity than their opponents. Our work differs from this model in that whenever the players have a low confidence level (about their opponents' intention), instead of refusing to play they can make use of the alternative, but provenly efficient strategy, of arranging prior commitments. As we have shown, this combination of the two strategic behaviors performs substantially better than the sole intention recognition one.

The key role of intention recognition in the current model is to allow choosing cooperative partners and avoid reliance on arranging a costly explicit commitment. In environments where partner selection is possible—that is, when people can choose with whom they associate for mutualistic endeavors—then implicit commitments are evolved, by which people behave as if they had bargained with others in order to reach an agreement, in accordance with contractualist moral psychology^32^,^33^. Hence, our results suggest that intention recognition might have been shaped by natural selection to enable effective partner selection, which in turn drives the evolution of implicit commitments, thereby avoiding the cost of arranging explicit commitments.

Several behavioral experiments on intention based strategies exist that are closely related to our model. The experiment in Ref. 26 uses a sequential PD (in the presence of noise) where the second-moving player can recognize the first-moving player's intention, and choose whether to punish a defecting act. The experiment showed that individuals tend to use strong punishment against those who are recognized to have a clear intention of defection while no (or weak) punishment is used against those who defected but the act is recognized to be unintentional. Our work differs from this experimental setting in that the intention recognition process is done prior to the interaction (to find out whether it is necessary to arrange prior commitments), while it is posterior in the experiment, i.e. after the move has been made. Another experiment in Ref. 21 showed that, in the course if the repeated Prisoner's Dilemma, if co-players' intention can be observed, it significantly fosters cooperation since unintentional defection caused by noise can be forgiven, as also shown theoretically in Ref. 22. Note that both experiments have been designed so that the intention recognition process is facilitated, thereby guaranteeing a high confidence level. In such cases, as shown in the present work, the synergy of intention recognition and commitments, both aiming at clarifying co-players' intention, can promote a high level of cooperation.

Several extensions to the present model can be described. In our model we have considered a general one-shot interaction scenario, but we envisage that as more prior experience is incorporated, for instance by observing direct or indirect past actions of the co-player, intention recognition can be performed better, thereby leading to better performance of IRCOM. Indeed, in Refs. 22, 34, in the context of the repeated PD with implementation noise, Artificial Intelligence based intention recognition strategies^35^,^36^ can more accurately assess a co-player's intention whenever more past interactions are taken into account. In SI, we consider a more effective IRCOM strategy, having a more accurate intention recognition capability (see Figure S3). Our numerical results show that, whenever the intention recognition model is efficient enough, the intention recognition strategy by itself alone (i.e. IRCOM with *θ* = 0) performs quite well, complying with the results obtained in Refs. 22, 34, where concrete intention recognition models are deployed.

Overall, our work indicates that, on the one hand, it is evolutionarily advantageous to be able to avoid arranging costly commitments whenever the co-player's intention can be assessed with sufficient confidence and accuracy. On the other hand, arranging prior commitments may be also unavoidable, depending on the strength of the dilemma, in order to reach a high level of cooperation.

## Methods

Our analysis is based on evolutionary game theory methods for finite populations^37^,^38^. In the context of evolutionary game theory, the individuals' or agents' payoff represents their *fitness* or social *success*. The dynamics of strategy change in a population is governed by social learning, that is, the most successful agents will tend to be imitated by the others. There are many ways to model social learning^5^,^39^,^40^. Adopting one of the most frequently used ones, we consider the so-called pairwise comparison rule^41^, which assumes that an agent *A* with fitness *f_A_* adopts the strategy of another agent *B* with fitness *f_B_* with probability given by
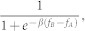
where *β* controls the ‘imitation strength', i.e., how strongly the agents are basing the decision to imitate on fitness comparisons. For *β* = 0, we obtain the limit of neutral drift – the imitation decision is random. For large *β*, imitation becomes increasingly deterministic.

In the absence of mutations, the end states of evolution are inevitably monomorphic: once such a state is reached, imitation cannot produce any change. We thus further assume that, with a certain mutation probability *μ* > 0 (also dubbed the exploration rate^42^), an agent switches randomly to a different strategy without imitating any other agent. The resulting Markov Chain has a stationary distribution, which characterizes the average time the population spends in each of these monomorphic end states. Yet, for arbitrary exploration rates and number of strategies, stationary distributions are often cumbersome to compute^43^,^44^,^45^.

Fortunately, in the case of small exploration or mutation rates, analytical computation of this stationary distribution can conveniently be computed^38^,^43^,^46^,^47^. The small exploration rates guarantee that any newly occurred mutant in a homogeneous population will fixate or become extinct long before the occurrence of another mutation. Hence, the population will always consist of at most two strategies in co-presence. This allows one to describe the evolutionary dynamics of our population in terms of a reduced Markov Chain, whose size is equal the number of strategies being considered, and each state represents a possible monomorphic end state of the population associated with a one of the strategies. The transitions between states are defined by the fixation probabilities of a single mutant of one strategy in a homogeneous population of individuals adopting another strategy^46^.

More precisely, let *N* be the size of the population. Suppose there are at most two strategies in the population, say, *k* agents using strategy A (0 ≤ *k* ≤ *N*) and (*N* − *k*) agents using strategy B. Thus, the (average) payoff of the agent that uses A or uses B can be written as follows, respectively,
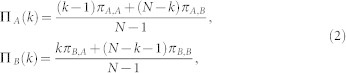
where *π_X_*_,*Y*_ stands for the payoff an agent using strategy *X* obtained in an interaction with another agent using strategy *Y*, given by the payoff matrix (9).

Now, the probability to change, by ±1, the number *k* of agents using strategy A at each time step can be written as
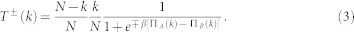
The fixation probability of a single mutant with a strategy A in a population of (*N* − 1) agents using B is given by^38^,^41^,^43^,^46^,^48^
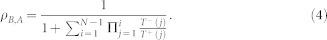
In the limit of neutral selection (*β* = 0), *T*^−^(*j*) = *T*^+^(*j*) µ*j*. Thus, *ρ_B_*_,*A*_ = 1/*N*. Considering a set {1, …, *q*} of different strategies, these fixation probabilities determine a transition matrix 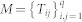, with *T_ij_*_,*j*≠*i*_ = *ρ_ji_*/(*q* − 1) and 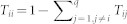, of a Markov Chain. The normalized eigenvector associated with the eigenvalue 1 of the transposed of *M* provides the stationary distribution described above^38^,^43^,^46^,^48^, describing the relative time the population spends adopting each of the strategies.

### Deriving Payoff Matrix

The one-shot Prisoner's Dilemma can be described with the following payoff matrix:
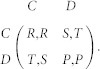
Once the interaction is established and both players have decided to play C or D (with or without commitment arrangements), both players receive the same reward *R* (penalty *P*) for mutual cooperation (mutual defection). Unilateral cooperation provides the sucker's payoff *S* for the cooperative player and the temptation to defect *T* for the defecting one. The payoff matrix corresponds to the preferences associated with the Prisoner's Dilemma when the parameters satisfy the ordering, *T* > *R* > *P* > *S*^5^,^49^. In the main text, we use the Donor game, a special case of the PD, with *T* = *b*; *R* = *b* − *c*; *P* = 0; *S* = −c, where *b* and *c* are the benefit and cost of cooperation, respectively.

When proposing commitment, the average payoff of IRCOM, as the row player, reads^27^
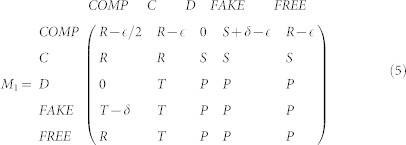
The probability that IRCOM relies on the intention recognition prediction, and the prediction was actually correct, can be written as joint probability distribution^50^
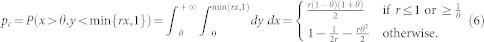
Similarly, the probability that IRCOM relies on the intention recognition prediction, but the prediction was not correct, is
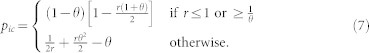
Hence, IRCOM cooperation probability when playing with another IRCOM player is, *θ* + *p_c_*.

The payoff matrix for IRCOM when relying on intention recognition reads
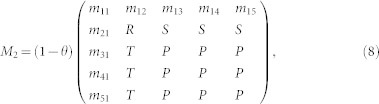
where
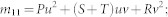

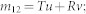

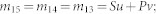

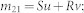

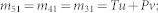

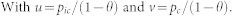
Finally, the payoff matrix for IRCOM (as a row player) reads
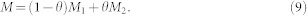


## Author Contributions

T.A.H., F.C.S., T.L. and L.M.P. designed the research. The models were implemented by T.A.H. Results were analyzed and improved by T.A.H., F.C.S., T.L. and L.M.P. T.A.H., F.C.S., T.L. and L.M.P. wrote the paper together.

## Supplementary Material

Supplementary Information Supporting Information

## Figures and Tables

**Table 1:** Variables and parameters used in the model

Symbols	Description
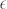	The cost of arranging a commitment deal
*δ*	The compensation cost
*c*	The cost of cooperation in the PD game
*b*	The benefit of cooperation in the PD game
*x*	The degree of confidence in a correct intention prediction
*θ*	The confidence threshold to rely on intention recognition (i.e. if *x* > *θ*)
*r*	The accuracy-to-confidence ratio
*y*	The accuracy of intention prediction, given the confidence (*y* = *r* × *x*)
*β*	The intensity of selection
