# Supplementary material for: Synergy between intention recognition and commitments in cooperation dilemmas
Source: Sci Rep. 2015 Mar 20;5:9312. doi: 10.1038/srep09312 (PMC4366807; doi:10.1038/srep09312)
Supplement: Supplementary Information — Supporting Information [file srep09312-s1.pdf]

Supporting Information: Synergy between intention recognition and commitments in cooperation dilemmas

The Anh Han[α], Francisco C. Santos[β,γ], Tom Lenaerts[δ,λ] and Luís Moniz Pereira[ϵ]

January 19, 2015

[α] School of Computing, Teesside University, Borough Road, Middlesbrough, UK TS1 3BA

[β] INESC-ID and Instituto Superior Técnico, Universidade de Lisboa, IST-Taguspark, 2744-016 Porto Salvo, Portugal

[γ] ATP-group, CMAF, Instituto para a Investigação Interdisciplinar, P-1649-003 Lisboa Codex, Portugal

[δ] AI lab, Computer Science Department, Vrije Universiteit Brussel, Pleinlaan 2, 1050 Brussels, Belgium

[λ] MLG, Département d'Informatique, Université Libre de Bruxelles, Boulevard du Triomphe CP212, 1050 Brussels, Belgium

[ϵ] NOVA Laboratory for Computer Science and Informatics (NOVA LINCS), Departamento de Informática, Faculdade de Ciências e Tecnologia, Universidade Nova de Lisboa, 2829-516 Caparica, Portugal

*corresponding author: T.Han@tees.ac.uk

In this supporting information, we provide additional numerical results to show the robustness of our conclusions in the main text.

1 Results for different benefit-to-cost ratios

In Figure S1 we show the cooperation level from commitment strategies, IRCOM and COMP, as a function of the cost of arranging commitment ϵ and the compensation cost δ, the improvement in cooperation level compared to the case where there is no IRCOM, and such an improvement in percentage. We also plot the same quantity for different b/c. In general, we observe that improvement is always possible, and furthermore, the larger b/c (i.e. the less harsh the PD), the larger the improvement is achieved.

Figure S2 shows the frequency of COMP and IRCOM (at the optimal confidence threshold) for different values of ϵ and δ, and for different b/c ratios. In general, for sufficiently large δ and low ϵ, IRCOM dominates the population. Interestingly, in contrast to COMP, it is not always the case that the frequency of IRCOM is smaller for larger ϵ. IRCOM is actually more frequent when ϵ is sufficiently high, which is larger for larger b/c.

2 More efficient intention recognition

In the main text we have used a very inefficient intention recognition model, where the accuracy of intention recognition is a random number derived from $[0, 1]$. It is not surprising that the performance of the intention recognition strategy solely—which corresponds to IRCOM with $\theta = 0$, is very poor. In the sequel, let us study the model using more efficient intention recognition models (Figure S3).

We consider that the prediction accuracy, Y, is randomly distributed in the interval $[\gamma, 1]$, where a larger γ reflects a more efficient intention recognizer at work. In an increasing order of efficiency, Y is uniformly drawn from intervals $[0, 1]$, $[0.3, 1]$, $[0.6, 1]$, and $[0.9, 1]$. Note that in the context of iterated interactions (e.g. in the framework of the iterated Prisoner's Dilemma), these levels of efficiency can be achieved (on average) by considering large enough numbers of

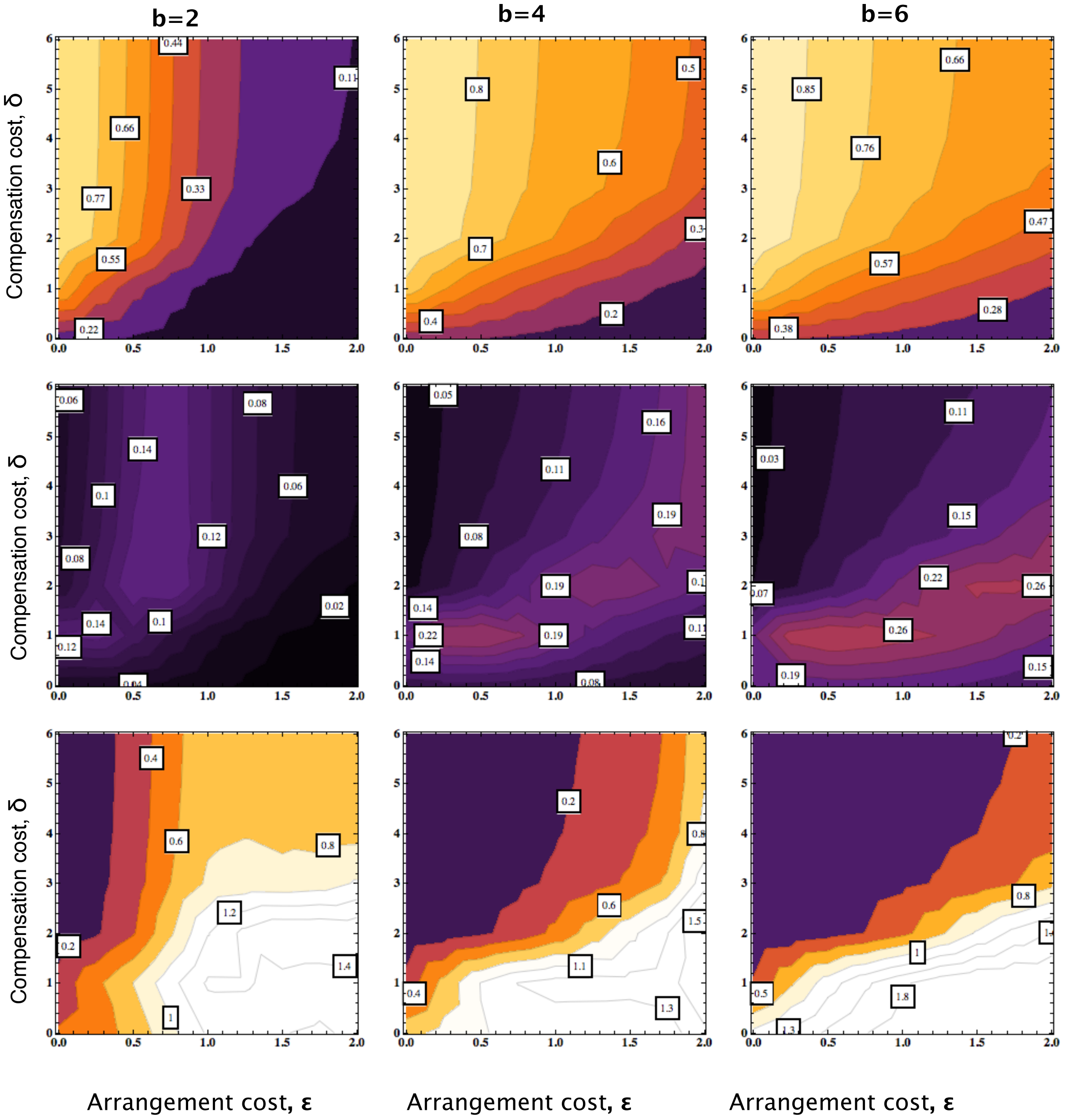

Figure S1: Cooperation level from commitment strategies, IRCOM and COMP, as a function of the cost of arranging commitment ϵ and the compensation cost δ (first row); Improvement in cooperation level compared to the case where there is no IRCOM (second row); and such an improvement in percentage (third row). We plot for different b/c. The larger b/c, the larger the improvement. Parameters: Panels (a) and (b): $\delta = 4$; $\epsilon = 0.7$; In all cases, $b = 2,\ c = 1$; $r = 1$; $N = 100$; $\beta = 0.1$.

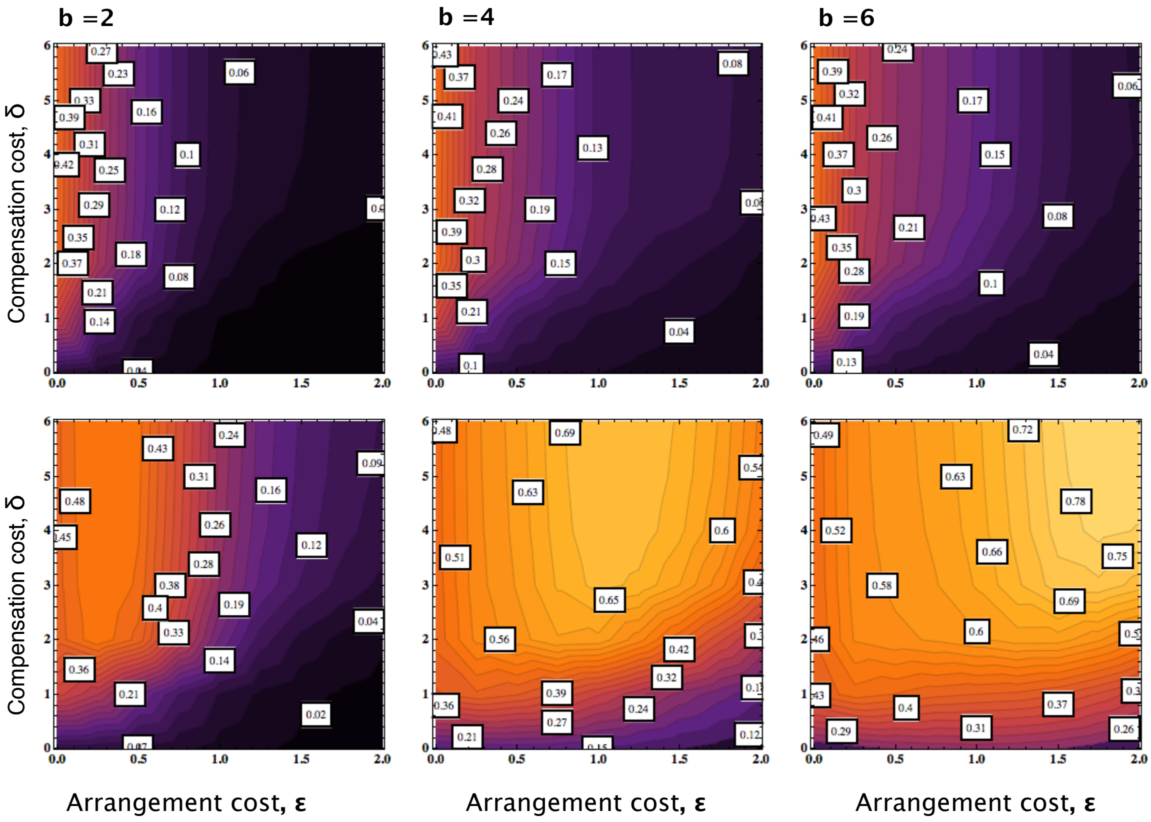

Figure S2: Frequency COMP in a population of five strategies COMP, C, D, FREE, and FAKE (top row) and of IRCOM (at optimal confidence threshold) in a population of six strategies IRCOM, COMP, C, D, FREE, and FAKE (bottom row) for varying ϵ and δ, and for different b/c. In general, for sufficiently large δ, IRCOM dominates the population for small ϵ. Interestingly, in contrast to COMP, it is not always the case that the frequency of IRCOM is smaller for larger ϵ. IRCOM is actually more frequent when ϵ is sufficiently large, which is larger for larger b/c. Parameters: In all cases: $r = 1$; $N = 100$; $\beta = 0.1$.

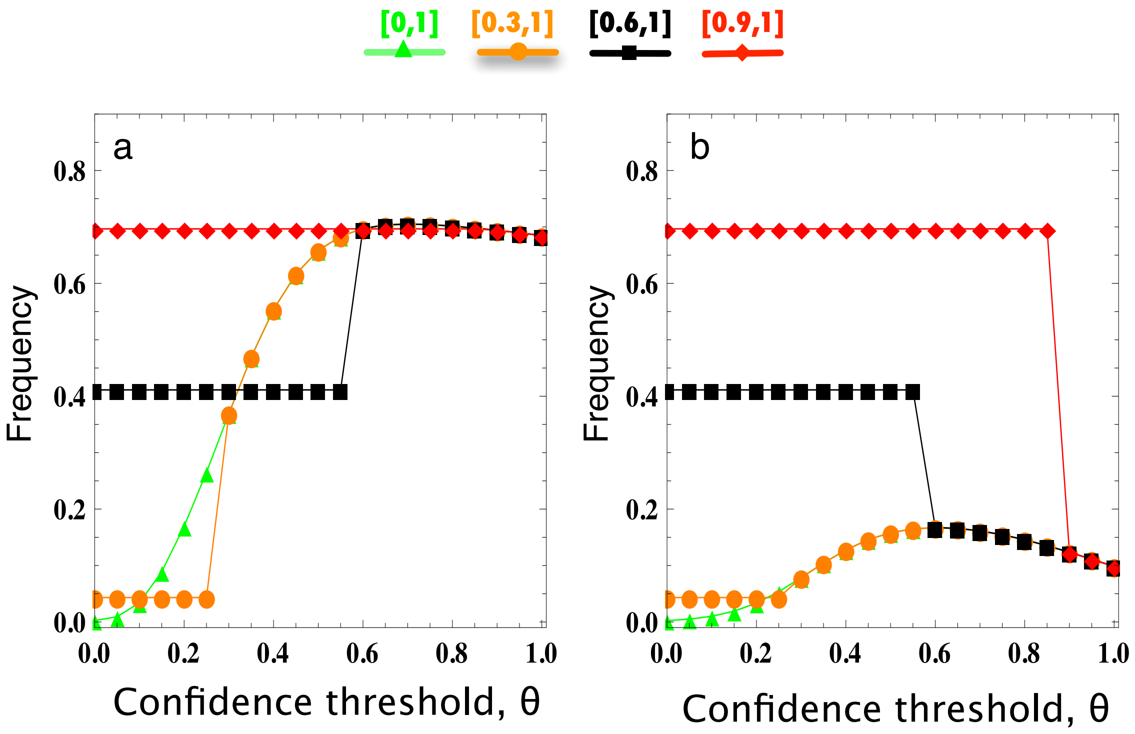

Figure S3: Frequency of IRCOM as a function of confidence threshold, θ, in a population of IRCOM, C, D, FAKE and FREE individuals. We consider different probability distributions of the intention prediction accuracy, reflecting the efficiency or precision of the intention recognition model at work. Namely, Y is uniformly drawn from $[\gamma, 1]$, with $\gamma = 0, 0.3, 0.6, 0.9$. The results show that when intention recognition is highly accurate, it is worth relying more on the intention predictions, even exclusively (see $\gamma = 0.9$ in panel a, and $\gamma = 0.6$ and 0.9 in panel b). Parameters: $\epsilon = 0.25,\ \delta = 4$ (panel a) and $\epsilon = 1,\ \delta = 2$ (panel b); payoff entries, $T = 2,\ R = 1,\ P = 0,\ S = -1$; accuracy over confidence ratio, $r = 1$; population size, $N = 100$; imitation strength, $\beta = 0.1$.

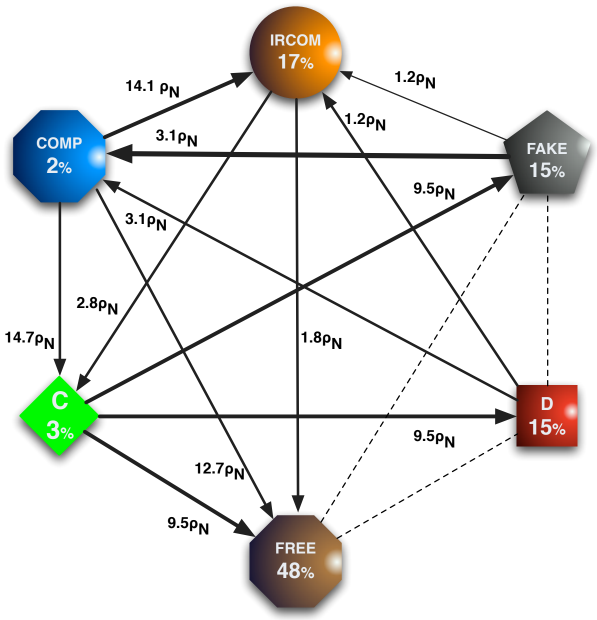

Figure S4: Transitions probabilities and stationary distributions for a large ϵ ($\epsilon = 3$). Other parameters similar to main text: $\delta = 4$, $r = 1$; $b = 4$, $c = 1$; $N = 100$; $\beta = 0.1$.

interactions between two players (or high enough probabilities of a next interaction[4,5]), given that the noise is small enough. Normally, the more an intention recognizer interacts with a fixed co-player, the better it predicts its co-player's intention. For example, this holds for the two intention recognition models described in[2,3]. Furthermore, in[1], the authors present experimental evidence showing that, in a one-shot PD, subjects of only brief acquaintance were able to recognize players with an intention to defect with more than twice chance accuracy.

The results show that, whenever the intention recognition model is efficient enough, the intention recognition strategy solely (i.e. IRCOM with $\theta = 0$) performs quite well, complying with the results obtained in[2,3], where concrete intention recognition models are deployed. However, when a quite strong commitment deal can be envisaged (Figure S3a), arranging it can still glean some evolutionary advantage. But in case that only weak commitment deals can be arranged (Figure S3b), it is then more beneficial to rely, even exclusively, on the intention recognition strategy should it be efficient enough.

References

[1] Frank, R. H., Gilovich, T., and Regan, D. T. 1993. The evolution of one-shot cooperation: An experiment. *Ethology and Sociobiology*, 14(4):247 – 256.

[2] Han, T. A., Pereira, L. M., and Santos, F. C. 2011. Intention recognition promotes the emergence of cooperation. *Adaptive Behavior*, 19(3):264–279.

[3] Han, T. A., Pereira, L. M., and Santos, F. C. 2012. Corpus-based intention recognition in cooperation dilemmas. *Artificial Life journal*, 18(4):365–383.

[4] Nowak, M. A. 2006. Five rules for the evolution of cooperation. *Science*, 314(5805):1560. DOI: 10.1126/science.1133755.

[5] Sigmund, K. 2010. *The Calculus of Selfishness*. Princeton University Press.
